# Creasote in Whooping Cough

**Published:** 1900-11-10

**Authors:** 


### CREASOTE IN WHOOPING COUGH.

Whooping cough, in the form in which it often comes before the practitioner, is a somewhat complicated malady, the importance of the paroxysm being overwhelmed by the severity of the accompanying bronchitis. In many cases, however, the paroxysm is everything, and but for its annoying repetition the sufferings of the little patient would not be great. Mr. Edward Godson,[1] of Manchester, has been endeavouring to ascertain from his medical friends what drugs they employ in the treatment of the paroxysmal stage, and which drugs are considered by them to give the best results. According to the answers received the drugs most commonly employed and chiefly depended on are antipyrin, belladonna, bromides, carbolic acid, creasote, and opium, the antispasmodics being always combined with expectorants, of which the alkalies are the greatest favourites. Inhalants also seem to be in general use. None of the answers received, however, were enthusiastic except from those who had used creasote vapour. The results obtained by this method were more satisfactory when the inhalation was continuous than when it was intermittent, and the simplest and best method seemed to be to sprinkle the drug on a cloth and hang the cloth in the nursery or sick-room to dry. In this way a highly impregnated air could be constantly supplied to the patient. The inhalation arranged in this way appeared to be free from danger except when the chest was full of moist sounds, when its effects should be carefully watched. Mr. Godson himself confirms the asserted advantage of belladonna in broncho-pneumonia.

[1] Brit. Med. Jour., Nov. 3.

